# Supplementary material for: TP5: A Novel Therapeutic Approach Targeting Aberrant and Hyperactive CDK5/p25 for the Treatment of Colorectal Carcinoma
Source: Int J Mol Sci. 2023 Jul 21;24(14):11733. doi: 10.3390/ijms241411733 (PMC10380212; doi:10.3390/ijms241411733)
Supplement: Supplementary file 1 [file ijms-24-11733-s001.zip › ijms-2450249-supplementary.pdf]

Supplementary Materials:

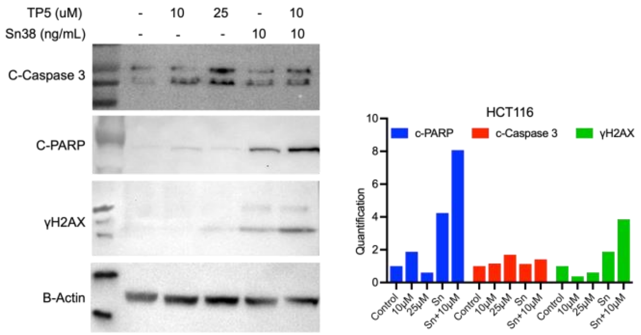

Supplementary Figure S1. Protein level of cleaved Caspase 3, cleaved PARP, and γH2A.X in HCT116 cells after 24h of treatment as indicated. TP5: 10 and 25 μM, Sn38: 10 ng/mL. Quantification of the blots is presented on the right side.

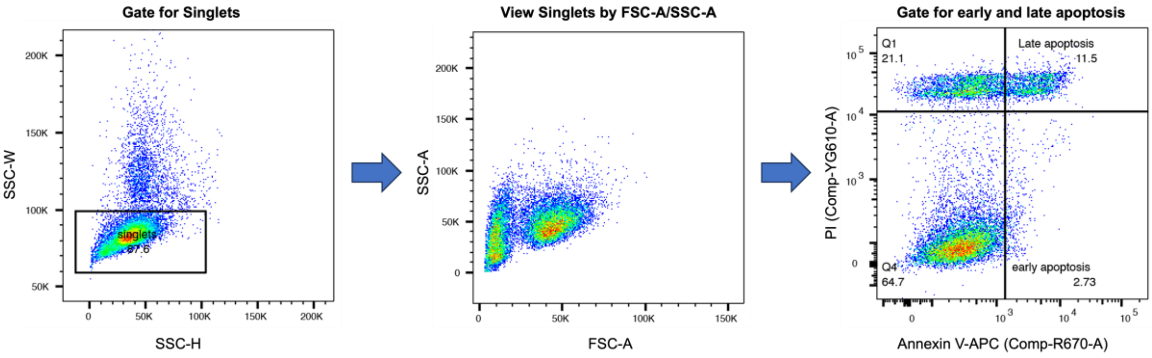

Supplementary Figure S2. Gating strategy for cell apoptosis analysis. (Left) Singlets were gated by SSC-H/SSC-W to exclude doublets. (Middle) Two major subpopulations were present in the singlets. The subpopulation with smaller FSC-A was also included for cell apoptosis analysis. (Right) Apoptotic cells were gated by Annexin V-APC (compensated-R670-A) and PI (compensated-YG610-A). Early apoptosis was defined as Annexin V+ PI-, and late apoptosis was defined as Annexin V+ PI+.
